# Missed Doses and Take-Home Dosing Patterns in Methadone and Buprenorphine Patients

**DOI:** 10.1192/bjo.2026.11532

**Published:** 2026-06-30

**Authors:** Mohammad Ali

**Affiliations:** Addictions Recovery Community (ARC-MK), Milton Keynes, United Kingdom

## Abstract

**Aims:**

Adherence to opioid substitution therapy is critical for treatment success, yet missed doses and unsupervised take-home doses can increase relapse or overdose risk. This service evaluation aimed to assess patterns of missed supervised doses and take-home medication use, identify factors associated with non-adherence, and evaluate potential service improvements.

**Methods:**

A retrospective service evaluation was conducted in an addictions psychiatry outpatient clinic. Adults aged ≥18 years receiving methadone or buprenorphine for at least three months were included. Patients were excluded if they had transferred care during the evaluation period, had incomplete pharmacy records, or were unable to engage in routine clinic follow-up due to acute intoxication, severe withdrawal, cognitive impairment, or acute psychiatric instability. Data were extracted from clinic and pharmacy records, including supervised dose attendance, take-home dosing patterns, demographic information, concurrent substance use, and housing status. Descriptive statistics were used to summarise missedsupervised doses, take-home medication issues, and associated factors, with exploratory comparisons between patients with and without polysubstance use.

**Results:**

A total of 120 patients on opioid substitution therapy (methadone n=75, buprenorphine n=45) were included in the service evaluation. Missed supervised doses were common, with 36 patients (30%) missing ≥2 doses in the preceding month and 18 patients (15%) missing ≥4 doses. Issues with take-home medication were documented in 22 patients (18%), including misplacement (n=12) and suspected diversion (n=10). Polysubstance use was prevalent, with 52 patients (43%) reporting concurrent use of cocaine, cannabis, or illicit benzodiazepines; missed supervised doses were more frequent among these patients (42% vs 21%, p<0.05). Younger age (<35 years), unstable housing, and high polysubstance use were associated with higher rates of missed doses. In 28 of the patients with frequent missed doses, additional adherence support was documented, including increased supervised dosing and motivational interventions. Review of clinic and pharmacy records demonstrated that systematic evaluation of adherence patterns is feasible and provides clinically actionable information for optimizing patient safety and treatment outcomes.

**Conclusion:**

Missed supervised doses and take-home medication issues are common among patients receiving opioid substitution therapy, particularly in those with polysubstance use, younger age, or unstable housing. These findings highlight the importance of structured adherence monitoring, targeted support interventions, and careful supervision of take-home doses to optimize treatment outcomes and reduce harm. Routine evaluation of adherence patterns is feasible in outpatient addiction services and can provide actionable insights for improving patient safety and the overall quality of care.

